# Silencing essential gene expression in *Mycobacterium abscessus* during infection

**DOI:** 10.1128/spectrum.02836-23

**Published:** 2023-10-13

**Authors:** Yves-Marie Boudehen, Yara Tasrini, John Jairo Aguilera-Correa, Matthéo Alcaraz, Laurent Kremer

**Affiliations:** 1 Centre National de la Recherche Scientifique UMR 9004, Institut de Recherche en Infectiologie de Montpellier (IRIM), Université de Montpellier, Montpellier, France; 2 INSERM, IRIM, Montpellier, France; Johns Hopkins University School of Medicine, Baltimore, Maryland, USA

**Keywords:** Tet-OFF system, gene repression, anhydrotetracycline, infection, *Mycobacterium abscessus*, zebrafish, MmpL3

## Abstract

*Mycobacterium abscessus* is a multi-drug-resistant non-tuberculous mycobacterial species causing tuberculosis-like lung infections. The functional characterization of the extensive repertoire of genes encoding virulence factors and drug targets has been largely restricted by the lack of powerful genetic tools. In this study, we evaluated the performances of a Tet-OFF system, previously optimized for *M. tuberculosis* and *M. smegmatis*. Fluorescence reporter *M. abscessus* strains were developed where mCherry was under the control of the Tet-OFF regulatory system on an integrative plasmid. The addition of anhydrotetracycline (ATc) correlated with a decrease in fluorescence intensity not only in solid and broth medium but also in colony biofilms, for both smooth and rough variants of *M. abscessus*. Next, unmarked *mmpL3* conditional knockdown mutants were engineered in both smooth and rough *M. abscessus*. Biochemical studies indicated that the addition of ATc was associated with a dose-dependent decrease in (i) the production of the MmpL3 protein, (ii) the biosynthesis of trehalose dimycolate and mycolylated arabinogalactan, (iii) bacterial viability on agar and liquid medium as well as in biofilms. Importantly, intravenous injection of ATc in zebrafish embryos infected with the rough *mmpL3* conditional mutant impeded bacterial growth and correlated with a significant gain in embryo survival. These results suggest that *mmpL3* is essential for *M. abscessus* growth *in vitro* and in the infected host, thus validating MmpL3 as an attractive drug target. Together, this study demonstrates the potential of ATc-dependent repression to modulate the expression of *M. abscessus* genes in the infected host.

**IMPORTANCE:**

*Mycobacterium abscessus* represents the most common rapidly growing mycobacterial pathogen in cystic fibrosis and is extremely difficult to eradicate. Essential genes are required for growth, often participate in pathogenesis, and encode valid drug targets for further chemotherapeutic developments. However, assessing the function of essential genes in *M. abscessus* remains challenging due to the limited spectrum of efficient genetic tools. Herein, we generated a Tet-OFF-based system allowing to knock down the expression of *mmpL3*, encoding the mycolic acid transporter in mycobacteria. Using this conditional mutant, we confirm the essentiality of *mmpL3* in planktonic cultures, in biofilms, and during infection in zebrafish embryos. Thus, in this study, we developed a robust and reliable method to silence the expression of any *M. abscessus* gene during host infection.

## INTRODUCTION

*Mycobacterium abscessus* is a non-tuberculous mycobacterium responsible for a large array of human diseases such as skin and soft tissue infections, disseminated disease, or chronic lung infections (1). It represents also an important pathogen in cystic fibrosis (CF) patients where it colonizes the lungs and is often associated with a more rapid decline in pulmonary function (2). *M. abscessus* can also be associated with biomaterial-related infections, where the main pathological mechanism involves biofilm formation (3, 4), a feature also reported in CF patients (5). Biofilm formation contributes also to bacterial persistence in harsh environments (6), rendering eradication extremely challenging.

*M. abscessus* can exist as two different phenotypes, either displaying a smooth (S) or a rough (R) colony morphotype, typified by the presence or absence of cell surface glycopeptidolipids (GPL), respectively (7 – 11). These two variants significantly differ in their virulence potential with the R strain demonstrating a more pathogenic and aggressive behaviour in mice (12), zebrafish (7 – 9), and CF patients (13). *M. abscessus* pulmonary diseases are very difficult to treat, owing to the intrinsic resistance of this pathogen to most antimicrobials, irrespective of the morphotype (1, 14). Thus, new chemical entities are urgently needed to populate the *M. abscessus* drug pipeline. This has recently stimulated *M. abscessus* drug discovery by prioritizing screening of advanced tuberculosis-active compounds for anti-*M*. *abscessus* activity (15), among which an inhibitor of the mycolic acid transporter MmpL3 has been identified (16).

Essential genes are central to the activity of most antibacterial agents and are among the most attractive targets for new therapeutic developments. By definition, essential genes are key players in bacterial growth and/or infection. While their biological activities remain very difficult to investigate, this can be achieved through the development of genetic tools permitting to silence gene expression and to generate conditional knockdown mutants. Gene manipulation has been widely developed during these last two decades, mainly in *M. tuberculosis* and *M. smegmatis*, through the generation of knockout mutants and the development of regulated expression systems, which have been instrumental to our understanding of pathogenic mechanisms and to demonstrate the essentiality of genes encoding drug targets. However, functional genomics tools are limited in *M. abscessus*, with mutagenesis approaches developed in other species often proving to be challenging to adopt in *M. abscessus* (17, 18). Despite these difficulties, recent studies have reported the successful use of CRISPR/Cas-based systems to inducibly silence the expression of targeted genes in *M. abscessus* (19). A previous study has evaluated the potential interest of a conditional gene expression approach using the TetR/PipOFF system, allowing to generate a *fadD32* knockdown mutant in both S and R variants of *M. abscessus* (20). While these systems have allowed studying the effect of gene downregulation *in vitro*, they have not been exploited to investigate the effect of gene silencing in *M. abscessus* during the infection process.

TetR-based systems have been widely used in mycobacteria to modulate gene expression and designed either to induce (Tet-ON) or repress (Tet-OFF) gene expression in the presence of anhydrotetracycline (ATc) (21 – 23). ATc crosses biological membranes and allows regulating gene expression in *M. tuberculosis*-infected cells (21) and in infected mice (24). Whereas similar regulatory systems are applicable to *M. abscessus* growing either *in vitro* or in the host remains to be addressed. Herein, a Tet-OFF switch in which transcription of the target gene is turned off by the addition of ATc, previously optimized for *M. tuberculosis* and *M. smegmatis* (22), was implemented for use in *M. abscessus* S and R variants. The design of reporter strains in which a fluorescence marker was placed under the control of the Tet-OFF regulatory system was successfully used to demonstrate an ATc-dependent reduction of fluorescence intensity, on agar plates, in planktonic cultures, and in colony biofilms. Subsequently, *mmpL3* conditional mutants were engineered to assess the essentiality of *mmpL3* in *M. abscessus in vitro*, in colony biofilms as well as in infected zebrafish embryos.

## RESULTS

### Tet-OFF system-mediated mCherry repression is effective in *M. abscessus*

**Fig 1:**
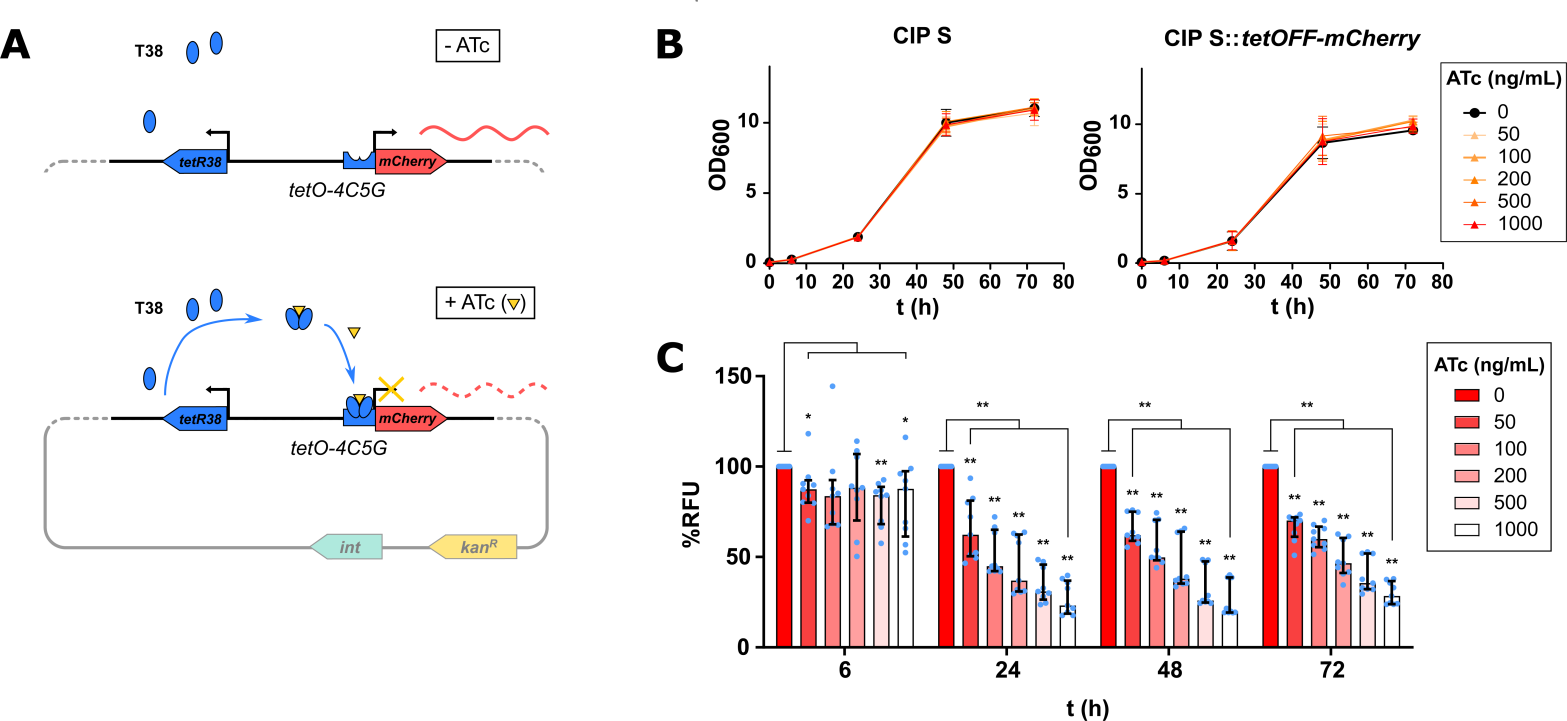
mCherry repression by ATc is effective in *M. abscessus*. (**A**) Schematic representation of the pMV306 derivative carrying the *mCherry* gene and the Tet-OFF system controlled by ATc. The absence of ATc allows constitutive gene transcription of *mCherry* (top), whereas transcription is blocked when ATc is present in the medium (bottom). (**B**) Growth of *M. abscessus* CIP S carrying the tetOFF-mCherry plasmid in the presence of ATc. Bacteria were grown in 7H9 complete medium supplemented or not with different ATc concentrations (50, 100, 200, 500, and 1,000 ng/µL) and optical densities of the cultures were recorded at 6, 24, 48, and 72 h. (**C**) Validation of mCherry repression in *M. abscessus* CIP S strain carrying repressible mCherry. Bacteria was grown in 7H9 complete medium supplemented or not with the indicated ATc concentrations. The fluorescence intensity of the mCherry signal in relative fluorescence units (RFU) was normalized to the cell density and expressed as % of control (0 ATc). *: *P*-value < 0.05; **: *P*-value < 0.01.

Tet systems have originally been developed for modulating gene expression in mycobacteria (23). To address whether this regulatory system is amenable for use *in M. abscessus*, we first constructed a vector derived from the integrative pMV306 where the *mCherry* gene was cloned downstream of the *tetO*-4C5G operator region, which is itself under the control of the T38 repressor (25), yielding the pMV306-TetOFF-mCherry. Repression of transcription is mediated by the addition of ATc, which binds to T38, allowing the T38-ATc complex to recognize the operator region, subsequently blocking transcription of the *mCherry* gene (Fig. 1A). This *mCherry* repressible plasmid was first introduced into the smooth (S) variant of *M. abscessus* CIP104536^T^. Growth of the parental (designated CIP S) and the transformed (designated CIP S::*tetOFF-mCherry*) strains was monitored in the presence of a wide range of ATc concentrations (50 to 1,000 ng/mL). Figure 1B shows that the presence of ATc did not alter bacterial growth, even at the highest concentration tested (1,000 ng/mL). Consecutively, the mCherry expression level was assessed by measuring the fluorescence intensity of the cultures exposed to the various ATc doses for 6, 24, 48, and 72 h (Fig. 1C). A dose-dependent reduction in fluorescence intensity was particularly evident from 24 h of incubation with ATc. The median repression rates were 87%, 83%, 88%, 84%, and 88% at 6 h and 38%, 55%, 63%, 69%, and 77% at 24 h for 50, 100, 200, 500, and 1,000 ng/mL ATc, respectively. However, longer exposure times to ATc did not further reduce fluorescence intensity, presumably because the half-life of ATc is around 20 h (26).

### Construction of a dual-fluorescent Tet-OFF reporter strain

**Fig 2:**
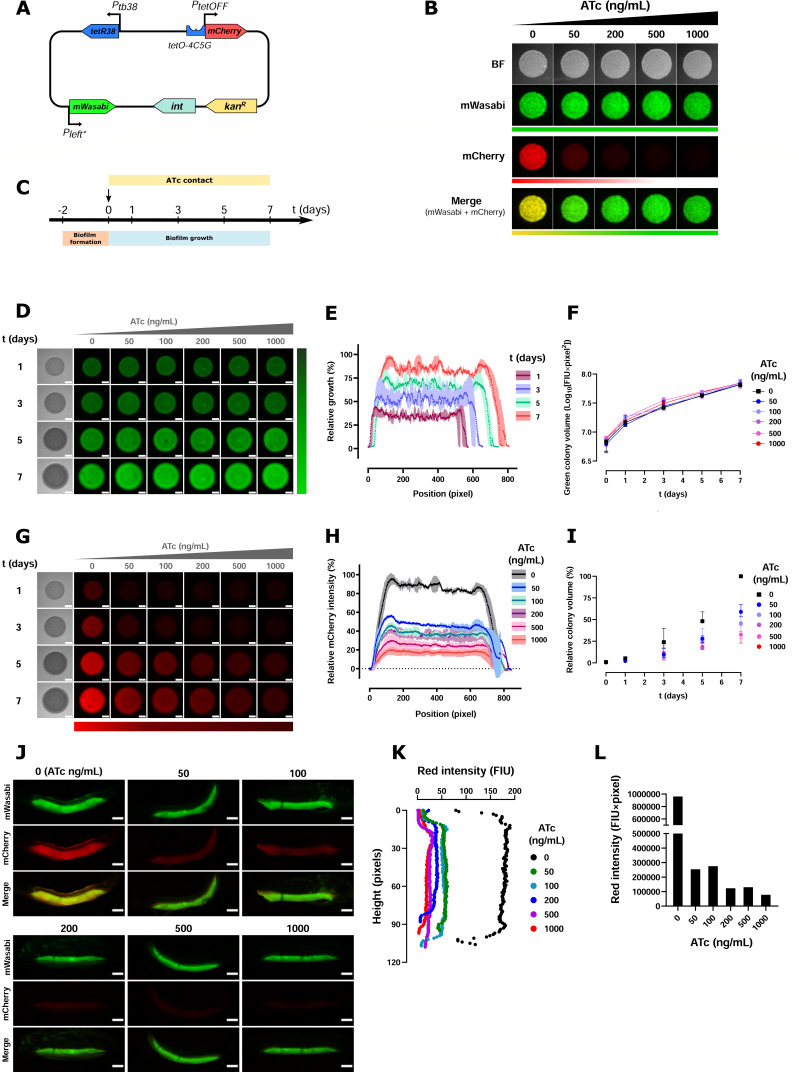
Use of dual-fluorescent reporter during *M. abscessus* smooth biofilm growth. (**A**) Schematic representation of the integrative pMV306 derivative encoding dual fluorescence. The nucleotide sequences of P_left*_ promoter and the *mWasabi* gene were introduced into the repressible mCherry plasmid version. (**B**) The *M. abscessus* CIP S strain, expressing repressible mCherry and constitutive mWasabi markers, was grown in 7H9 medium at OD = 1. Cultures were spotted onto LB agar plates supplemented or not with different concentrations of ATc (50, 100, 200, 500, and 1,000 ng/mL). Plates were incubated 3 days at 37°C and then fluorescent pictures were taken. (**C**) Representative timeline used for biofilm growth of *M. abscessus* CIP S expressing the dual-fluorescent reporter. Fluorescent pictures of the biofilms were taken at 1, 3, 5, and 7 days showing (**D**) mWasabi and (**G**) mCherry expression depending on ATc (50, 100, 200, 500, and 1,000 ng/mL). Scale, 2 mm. (**E**) Relative growth (expressed in percentage) of the untreated biofilm (ATc 0) measured by taking the average intensity levels of the constitutive mWasabi fluorescent protein along the diameter of biofilms. 100% growth is the maximum intensity at day 7. Light colors are corresponding to error bars for each pixel. (**F**) Green colony volume of S biofilms grown in the presence of different concentrations of ATc. (**H**) Intensity levels of mCherry fluorescent protein along the diameter of biofilms were retrieved from biofilms growing on different concentrations of ATc at day 7. The mCherry intensity was normalized to the control without drug (ATc 0) and expressed as %. (**I**) Relative colony volume percentage of S biofilms grown in the presence of different concentrations of ATc. Percentages were estimated by considering the red colony volume of S biofilms grown in the presence of 0 ng/mL at day 7 as 100%. (**J**) Transversal cuts of S biofilms grown in the presence of different concentrations of ATc at day 7. Scale, 2 mm. (**K**) Intensity levels of the mCherry fluorescent protein along the height of biofilms were retrieved from biofilms growing on different concentrations of ATc. (**L**) Total red fluorescence intensity over colony height. Fluorescence intensity was estimated for the products of each fluorescence intensity units (FIU) and its corresponding height (pixels).

To simultaneously follow mycobacterial growth and monitor gene repression in more complex situations, the *mWasabi* coding sequence placed under the control of the constitutive promoter P_left*_ (27) was introduced in the pMV306-TetOFF-mCherry (Fig. 2A). Following transformation into the S and R variants of *M. abscessus,* the expression of mWasabi and mCherry was determined by spotting bacterial cultures on Luria-Bertani (LB) agar containing ATc (50, 200, 500, and 1,000 ng/mL). While bacterial spots exhibited both red and green fluorescence in the absence of ATc (Fig. 2B; Fig. S1A), the addition of ATc into the medium was associated with a strong decrease of red fluorescence, without interfering with green signal. This confirms the ATc-dependent repression of mCherry expression and the constitutive production of mWasabi in both S and R variants on standard agar plates.

While only the R variant has a high propensity to aggregate *in vitro* and forms serpentine cords (7, 11), both S and R variants can produce biofilms under particular growth conditions. Biofilms are known to contribute to bacterial persistence and are more tolerant to antibacterial agents (28). We thus monitored colony-biofilm formation (29) using an experimental protocol illustrated in Fig. 2C, based on the rapid quantification of both green and red fluorescence levels. This approach allowed to determine the level of ATc penetration (reflected by the decline in mCherry expression) in these biofilm structures over time, while mWasabi expression remained unaffected by ATc (Fig. 2D). Monitoring mWasabi fluorescence indicated that the S variant grew horizontally and vertically (Fig. 2E), which is mirrored by the estimated colony volume for each concentration of ATc tested (Fig. 2F). In contrast, drop in mCherry fluorescence was ATc- and time dependent (Fig. 2G), with an optimal decrease in mCherry expression observed on day 7 (Fig. 2H). mCherry expression in S biofilms was negatively correlated with the ATc concentration from day 3 to day 7 (Fig. 2I) (ρ = −0.5486, *P*-value = 0.0184 for day 3; ρ = −0.7744, *P*-value = 0.0002 for day 5; and ρ = −0.9142, *P*-value <0.0001 for day 7). In addition, mCherry levels were also observed on cross-sections of the height of S biofilms (Fig. 2J). Quantification indicates that fluorescence intensity units decreased along the whole height in the presence of increasing ATc doses (Fig. 2K and L).

Assessing the evolution of fluorescence intensity in the R strain carrying the dual-reporter Tet-OFF construct showed that this strain grew mainly horizontally as compared to the S strain (Fig. S1B and C; Fig. 2C). Growth was also determined by estimating the colony-biofilm volume for each tested concentration of ATc over time (Fig. S1D). The red fluorescence did show an ATc- and time dependence (Fig. S1E). Optimal mCherry fluorescence decrease was observed at day 7 (Fig. S1F). The mCherry expression showed a negative correlation with the ATc concentration at days 3, 5, and 7 (Fig. S1G) (ρ = −0.6113, *P*-value = 0.0070 for day 5; and ρ = −0.732, *P*-value = 0.0005 for day 7).

### Generation and functional validation of an *mmpL3* conditional knockdown mutant

**Fig 3:**
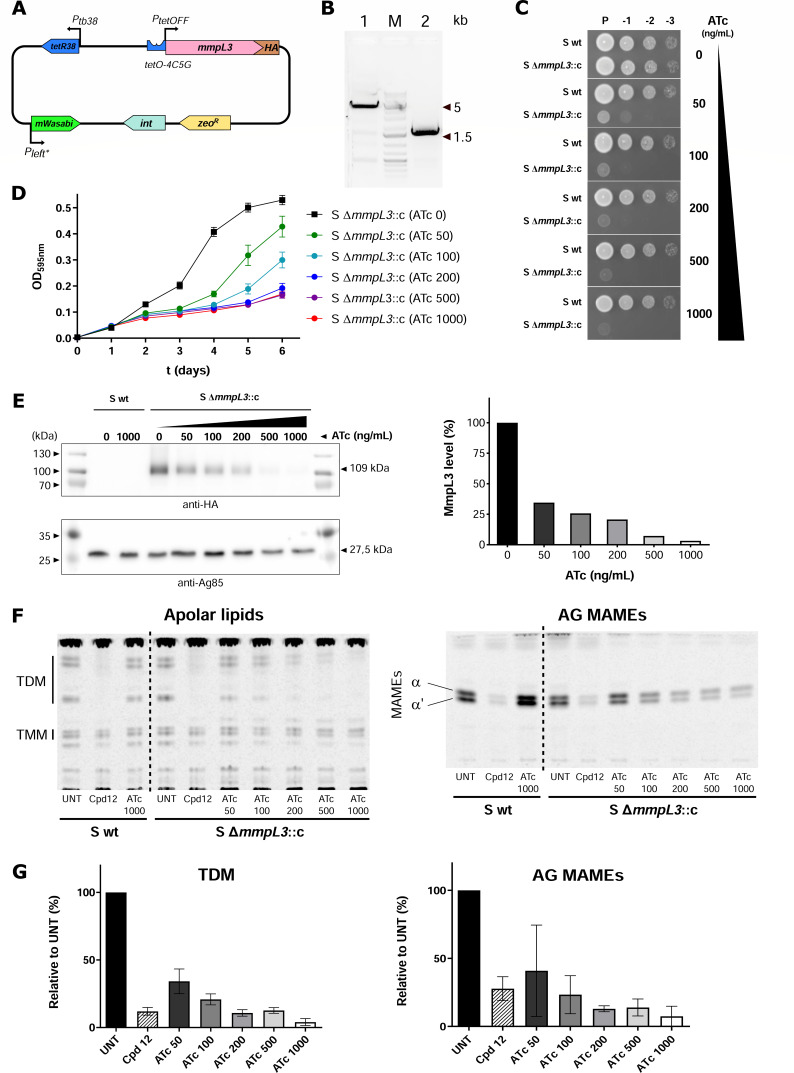
*In vitro* characterization of *M. abscessus mmpL3* conditional knockdown mutant. (**A**) Schematic representation of the pMV306 derivative carrying an HA-tagged *mmpL3* version under the control of the Tet-OFF system. (**B**) Confirmation of *mmpL3* wild-type locus deletion. PCR was done with primers producing a 4.5-kb fragment in wild-type (WT) (lane 1) and a 1.5-kb fragment for a deletion of *mmpL3* (lane 2). The 1.5-kb fragment was verified by Sanger sequencing. M, molecular size marker. (**C**) Parental (S wt) and *mmpL3* conditional mutant (S Δ*mmpL3*::c) cultures were grown to exponential phase, and 3 µL of 10-fold serial dilutions were spotted onto LB agar medium supplemented or not with ATc (50, 100, 200, 500, and 1,000 ng/mL). Plates were incubated at 37°C. Pictures were taken after 3 days. (**D**) Growth curves of the S *mmpL3* conditional mutant (S Δ*mmpL3*::c) were exposed to a range of ATc concentrations for 6 days. Optical density (595 nm) measurements were taken every day. (**E**) Western blots showing MmpL3 protein expression in the *mmpL3* conditional mutant strain (left panel). Bacteria were grown during 72 h in Sauton’s medium in the presence of increasing ATc concentrations, as indicated. The upper membrane was treated for HA immuno-detection and the lower membrane was treated for Ag85 immuno-detection (loading control). M, molecular size marker. MmpL3 protein levels quantified from Western blot (right panel). Quantification was normalized and expressed in % of the non-treated condition (ATc 0). Data are representative of two independent experiments. (**F**) Effect of ATc on mycolic acid transfer in the *mmpL3* conditional mutant strain. Apolar lipids were extracted and loaded to visualize trehalose monomycolate (TMM)/trehalose dimycolate (TDM) in the left panel. Arabinogalactan (AG)-coupled mycolic acids are shown in the right panel. A culture treated for 1 h with the indole-2-carboxamide compound 12 (Cpd12) was included as a positive control. The conditional mutant was compared to the parental strain left untreated (UNT) or treated with the highest ATc concentration (ATc 1000). (**G**) Densitometric analysis of TDM (left panel) and AG-bound mycolic acids (right panel) from the extracted lipids analyzed by thin layer chromatography relative to the untreated condition. Results are representative of three independent experiments.

*MmpL3* conditional mutants using the Tet system were successfully used to demonstrate *mmpL3* gene essentiality in *M. tuberculosis* (Δ*Rv0206c-mmpL3^Mtb^* using the TetR/Pip-OFF system) (30) as well as in *M. smegmatis* (Δ*MSMEG0250-mmpL3^Msm^* using the acetamide-inducible system) (31), implying that MmpL3 represents an attractive pharmacological target in these species. Subsequent work from various groups identified a wide panel of chemical entities inhibiting MmpL3 in *M. abscessus* (16, 32 – 36), although the essentiality of *mmpL3* in *M. abscessus* remains to be established. To do so, the *mCherry* coding sequence was replaced by an influenza hemagglutinin (HA)-tagged *mmpL3* version into the dual-reporter plasmid, generating the pMV306-TetOFF-*mmpL3-HA* (Fig. 3A) and introduced into the wild-type *M. abscessus* S strain. This merodiploid strain was then used to delete the endogenous *mmpL3* gene (Fig. 3B), resulting in a mutant whereby the unique copy of *mmpL3* was under the control of the Tet-OFF regulatory system. This conditional mutant was designated S Δ*mmpL3*::c, while the corresponding mutant in the R background was designated R Δ*mmpL3*::c. Essentiality of *mmpL3* was next investigated by spotting culture droplets of the conditional knockdown mutants onto agar plates containing range concentrations of ATc. In the absence of ATc, the growth of S Δ*mmpL3*::c and R Δ*mmpL3*::c strains was similar to their corresponding parental strains (Fig. 3C; Fig. S2A and C). Pronounced growth inhibition was observed in the presence of 50 ng/mL ATc, as evidenced by the lack of colonies at the 10^−2^ and 10^−3^ dilutions. At 200 ng/mL ATc (and higher concentrations), no colonies grew at the 10^−1^ dilution, and only residual growth was observed in the undiluted spots. This indicates that *M. abscessus* is unable to grow upon *mmpL3* transcriptional repression and suggests that *mmpL3* is essential for *in vitro* growth. These results were also confirmed in planktonic cultures for both S and R conditional knockdown mutants exposed for 6 days to increasing ATc concentrations (Fig. 3D; Fig. S2B). The planktonic growth showed a negative correlation with the concentration of ATc from day 2 onward for the S variant (ρ = - 0.576, *P*-value <0.0001 for day 2; ρ = - 0.631, *P*-value <0.0001 for day 3; ρ = - 0.743, *P*-value <0.0001 for day 4; ρ = - 0.819, *P*-value <0.0001 for day 5; and ρ = - 0.824, *P*-value <0.0001 for day 6). Similar results were also observed in the R variant (ρ = - 0.637, *P*-value <0.0001 for day 2; ρ = - 0.850, *P*-value <0.0001 for day 3; ρ = - 0.778, *P*-value <0.0001 for day 4; ρ = - 0.579, *P*-value <0.0001 for day 5; and ρ = - 0.321, *P*-value <0.0001 for day 6).

Transcriptional repression of *mmpL3-HA* by ATc was next assessed at the protein level by Western blotting using anti-HA antibodies. MmpL3-HA production declined in an ATc concentration-dependent manner (ρ = - 1, *P*-value = 0.0028). In the presence of 1,000 ng/mL ATc, MmpL3-HA expression was almost completely abolished, while expression of the antigen 85 (Ag85) remains stable (Fig. 3E). Because MmpL3 transports trehalose monomycolate (TMM) across the inner membrane, where it is used as a substrate by the Ag85 complex to convert TMM into trehalose dimycolate (TDM) and to esterify arabinogalactan (31, 37), we next investigated the effect of ATc-mediated loss of MmpL3 production on the synthesis of the cell wall lipids. ^14^C-acetate-labeled cultures were treated with ATc, and apolar lipids were extracted and separated by thin layer chromatography (TLC). Figure 3F (left panel) shows a dose-dependent inhibition of TDM synthesis, which was nearly abolished in the presence of 1,000 ng/mL ATc in S Δ*mmpL3*::c while remaining unaltered in the parental S progenitor. The effect of TDM synthesis inhibition in the ATc-mediated *mmpL3* knockdown mutant was comparable to chemical inhibition of MmpL3 in the wild-type S strain using the indole-2-carboxamide compound 12 (Cpd12), a known MmpL3 inhibitor (32). As expected, exposure to ATc of the *mmpL3* knockdown mutant, but not of the parental S strain, resulted in reduced mycolylated arabinogalactan levels, similar to the treatment with compound 12 (Fig. 3F, right panel). Quantifications of the ATc-dependent decrease in TDM and AG-bound mycolic acid levels in S Δ*mmpL3*::c are shown in Fig. 3G.

### Effect of *mmpL3* knockdown in rough colony biofilms

R colony biofilms of the *mmpL3* conditional mutant showed an ATc-dependent growth and size phenotype. The inhibitory effect of ATc is directly reflected by the colony diameter and particularly evident at day 7 (Fig. S3A). Indeed, untreated biofilm presents a maximal diameter of ~1,500 pixels, whereas the diameter of the biofilm treated with 1,000 ng/mL ATc was ~1,200 pixels (Fig. S3B). The biofilm growth showed a negative correlation with the concentration of ATc between day 3 and 7 (Fig. S3C) (ρ = - 0.5737, *P*-value = 0.0128 for day 3; ρ = - 0.749, *P*-value = 0.0003 for day 5; and ρ = - 0.826, *P*-value <0.0001 for day 7). The number of colony-forming units (CFU)/membrane at day 7 is negatively correlated with the concentration of ATc (ρ = - 0.7054, *P*-value = 0.0011) (Fig. S3D). Moreover, the colony density at day 7 presents a moderate negative correlation with the concentration of ATc (ρ = - 0.5988, *P*-value = 0.0086) (Fig. S3E). However, this effect was not observed with the S Δ*mmpL3*::c mutant (data not shown).

### *MmpL3* knockdown impairs bacterial growth and pathogenicity of *M. abscessus* in zebrafish

**Fig 4:**
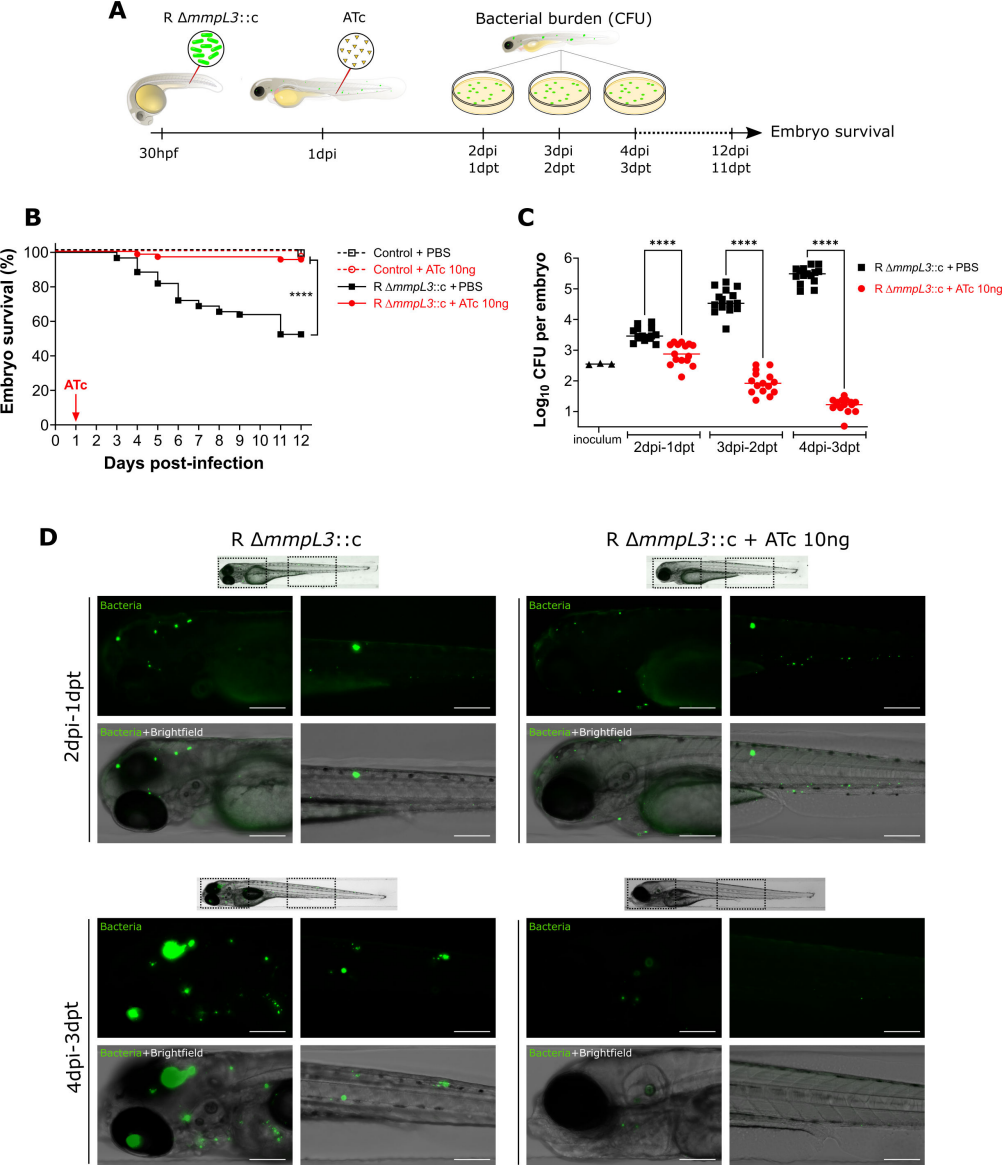
Conditional knockdown of *mmpL3* in infected zebrafish embryos leads to reduced bacterial burden and attenuation in virulence. (**A**) Schematic representation of the experimental design. (**B**) Survival of zebrafish embryos upon infection at 1 dpf with an average of 355 ± 18 CFU of R Δ*mmpL3*::c and treatment with 10 ng of ATc intravenously at 1 dpi. Embryos were monitored for 12 days. Pooled results from three independent experiments are shown (*n* = 24 per group per experiment). The red arrow represents the day of ATc injection. For each experiment, control groups were included for the infection as well as the treatment and are represented by dashed lines. Statistical analysis was performed using a log rank (Mantel cox) test. ****: *P* < 0.0001 . (**C**) Bacterial burden was analyzed using CFU counts at 2 dpi, 3 dpi, and 4 dpi. Inoculum was determined *a posteriori* and shown as a triangle. Each point represents an embryo, with rounds corresponding to untreated embryos and squares to treated embryos. Pooled results from three independent experiments are shown (*n* = 5 per group per time point per experiment). Horizontal lines correspond to the median. Statistical analysis was performed using one-way ANOVA with Šidák’s multiple comparisons test. ****: *P*-value < 0.0001. (**D**) Real-time imaging of zebrafish embryos infected with R Δ*mmpL3*::c expressing mWasabi, untreated (left panels) or treated with 10 ng of ATc (right panels). Panels show one representative embryo of each condition at 2 dpi-1 dpt (upper panels) and 4 dpi-3 dpt (lower panels). For each embryo, two squares with dotted lines indicate the fields imaged with an objective with higher magnification (head on the left and tail region on the right). Scale bars represent 200 µm.

Zebrafish embryos have been previously exploited to study the virulence of *M. abscessus* (7, 38, 39). Here, we used this model to determine if we could trigger *mmpL3* knockdown *in vivo* by treating intravenously infected embryos with ATc to assess the effect on the infection outcome (Fig. 4A). While treatment of zebrafish embryos with compounds is often done by simply adding the compound to the embryo water, our preliminary data indicate it is more reproducible to deliver the compound by microinjection instead of immersion (data not shown). Therefore, we first evaluated the toxicity of ATc microinjection at 2 days post-fertilization (dpf) using an increasing dose ranging from 1 ng to 100 ng and monitored treated embryos for 11 days. We found no toxicity signs in embryos treated from 1 to 10 ng of ATc, as opposed to embryos treated with 20 ng, which started to exhibit toxicity signs increasing with 50 ng and 100 ng (Fig. S4; Table S4). We, thus, opted for 10 ng of ATc which was the highest safest dose. We then injected 30 h post-fertilization (hpf) embryos in the caudal vein with 355 ± 18 CFU of R Δ*mmpL3*::c, which were subsequently treated at 1 day post-infection (dpi) with 10 ng ATc to repress the expression of *mmpL3* (Fig. 4A). Daily monitoring of embryo survival showed an increase in mortality over time in the non-treated group (53% mortality at 12 dpi) as expected, while only 5% of embryos treated with ATc succumbed to the infection (Fig. 4B). This strongly suggests that the temporal control of *mmpL3* repression is working *in vivo*. In agreement with the reduced embryo mortality, the bacterial burden from ATc-treated embryos was linearly decreasing over time (*r* = −0.9105; *P*-value <0.0001) at a rate of −0.8418 log_10_ per day (*P*-value <0.0001), as opposed to the non-treated control group in which the bacterial burden was linearly increasing over time (*r* = 0.9340; *P*-value <0.0001) at a rate of 0.9674 log_10_ per day (*P*-value <0.0001) (Fig. 4C). Moreover, to corroborate the CFU results, we took advantage of the transparency of the embryos to monitor the evolution of the mWasabi fluorescence which reflects the bacterial burden. Real-time imaging of embryos clearly showed in one hand, an increase of green fluorescence over time in untreated embryos characterized with the presence of big infection foci (Fig. 4D left panels), and in another hand, a reduction of green fluorescence over time between 1 day post-treatment (dpt) and 3 dpt in treated embryos with only small residual infection foci persisting (Fig. 4D right panels).

Overall, these results correlate with the *in vitro* growth inhibition upon ATc treatment, confirm the use of the Tet-OFF system to modulate gene expression in *M. abscessus* in the infected host, and support the essential character of MmpL3 as a valuable drug target for the treatment of *M. abscessus* diseases.

## DISCUSSION

Deciphering the importance and biological functions of essential determinants in *M. abscessus* remains challenging due to the lack of relevant genetic systems to knockdown gene expression, despite the recent development of CRISPR/Cas-based systems to inducibly silence the expression of targeted genes in this species (19). In this study, we provided evidence that the Tet-OFF regulatory system is applicable to *M. abscessus* growing *in vitro* as well as in the infected host. First, we demonstrated that transcription of the target gene encoding a fluorescence marker is turned off in *M. abscessus* by the addition of ATc, as previously shown for *M. tuberculosis* and *M. smegmatis* (22). The ATc-dependent reduction of fluorescence intensity was directly observed on bacterial colonies and quantified in broth cultures as well as in colony biofilms, thus validating the efficacy and robustness of the method in *M. abscessus*. In addition, we showed that the Tet-OFF silencing system is functional in both S and R variants, which responded similarly to ATc, thus indicating that the presence of the outermost GPL layer in the S form did not play a major role in preventing/limiting the entry of ATc inside the bacilli. This prompted us to design an *mmpL3* conditional mutant in S and R variants to assess the essentiality of *mmpL3* in *M. abscessus*. In *in vitro* experiments, we found that growth inhibition was ATc dose-dependent, which could be directly linked to the decreased production of the MmpL3 protein, translating into the blockage of TMM transport. The lack of TMM export was related to reduced levels of AG mycolylation and reduced pools of TDM, an expected phenotype relying on the fact that TMM serves as a substrate for the Ag85 mycolyltransferases. We note that, even at the highest concentrations of ATc tested (500 and 1,000 ng/mL), both S Δ*mmpL3*::c and R Δ*mmpL3*::c grew very slowly in liquid media, presumably indicating that the promoter is leaky, as judged by the low levels of MmpL3 produced under these repressive conditions, which translates into a partial inhibition of TDM production and AG mycolylation. Nevertheless, despite the presence of residual levels of MmpL3 upon ATc-induced repression, these results highlight the vulnerability of MmpL3 and the ensuing implications for future chemotherapeutic developments for *M. abscessus* infections, as exemplified by the wide list of chemotypes inhibiting this transporter in *M. abscessus* (16, 32 – 36). Recent sequencing of a saturated transposon library in *M. abscessus* provided a comprehensive prediction of essential genes in this mycobacterial species (40). However, these essential predictions require now further experimental confirmation. Typically, the Tet-OFF system reported here can be extended for conditional gene modulation of any essential gene in *M. abscessus*. This will be particularly useful to confirm these predictions and investigating whether inhibiting essential proteins can provide therapeutic benefit, particularly warranted in the context of multiple drug resistance of *M. abscessus* and unmet medical need to control/eradicate *M. abscessus* infections (1, 14).

Biofilm development is one crucial pathogenicity factor in *M. abscessus* (41). Here, we demonstrated that the Tet-OFF system may be applied to the recently developed *M. abscessus* colony-biofilm model (42). This biofilm model consists of inducing bacterial colony growth onto a polycarbonate membrane laying on an agar plate (43), for which nutritional or antibiotic supplementation can be modified at will (29). We show here that ATc not only penetrates the different mycobacterial layers that compose the colony biofilms but that it can also modulate the expression of mCherry. These results are in line with a previous study reporting the enhanced accumulation of tetracycline in bacterial biofilms (44). The overexpression of hundreds of genes in *M. abscessus* biofilms has recently been documented in both minimal medium (45) and synthetic CF medium (46). Our results support the use of the Tet-OFF system for switching off these genes, providing a valuable approach to study the consequences of silencing these genes during biofilm development. Of note, the colony-biofilm model reported here does not recapitulate the cellular characteristics with respect to cell-substratum and cell-cell adhesion found for instance in mycobacterial pellicles growing at the liquid/air interface. Therefore, the differences observed in S and R colony biofilms may result from the absence of GPL in the R morphotype since their presence, in *M. smegmatis,* favors biofilm formation by facilitating cellular attachment to the substratum, sliding motility and intercellular aggregation (47, 48). To illustrate the feasibility of this method, we monitored the effect of silencing *mmpL3* gene expression during *M. abscessus* colony-biofilm development using R Δ*mmpL3*::c. Knocking down *mmpL3* gene expression reduced biofilm growth in an ATc-concentration-dependent manner that could be corroborated at the level of the relative colony volume over time. Interestingly, the negative correlation between density (CFU/colony volume) and ATc concentration at day 7 points to a decrease in mycobacterial viability within the biofilm. These results are reminiscent of the bactericidal effect of an *N*-substituted indolemethylamine (IMA6) targeting MmpL3, previously described on nutrient-starved and oxygen-depleted non-replicating *M. abscessus* biofilm (49).

A major finding of this work relies on the possibility to decrease the transcription level of *M. abscessus* genes during systemic infection in zebrafish embryos and visualize the effect of gene silencing at a spatiotemporal level in a living vertebrate. In the case of infection with R Δ*mmpL3*::c, intravenous injection of ATc at 1 dpi resulted in a rapid and progressive reduction in the bacterial loads (quantified by determining the CFU over time) and infection foci (observed by fluorescence imaging of whole embryos). This significantly rescued the survival of embryos, which barely showed signs of pathology, as compared with the non-treated group of animals. Strikingly, the phenotypes observed here in zebrafish using the inducible *mmpL3* silencing system were very similar to those observed following chemical inhibition when exposing *M. abscessus*-infected embryos to MmpL3 inhibitors, such as PIPD1 (16) or benzimidazole derivatives (34). Overall, these results are in line with a previous work demonstrating that MmpL3 is required for the replication and viability of *M. tuberculosis*, both under standard laboratory growth conditions and during the acute and chronic phases of infection in mice (50). While zebrafish embryos recapitulate the acute stages of *M. abscessus* infection in the sole context of innate immunity (7, 51), adult fish, where both innate and adaptive immunity are operative, have been shown to be useful to describe *M. abscessus* chronic infection (52). Future studies should address the applicability of ATc-induced gene silencing in adult zebrafish to investigate the biological role of both essential and non-essential genes in *M. abscessus* persistence and chronic infections.

To conclude, we have described a robust and stable genetic system to downregulate genes in *M. abscessus* under *in vitro* laboratory conditions, and our data highlight MmpL3 as an essential lipid transporter in *M. abscessus,* validating MmpL3 as target of therapeutic interest. This provides a strong incentive for the subsequent developments of new or improved MmpL3 inhibitors in the fight against *M. abscessus* pulmonary diseases.

## MATERIALS AND METHODS

### Bacterial strains, plasmids, and primers

Strains, plasmids, and primers are listed in Tables S1 to S3, respectively.

### Culture conditions

*Escherichia coli* strains Stellar (Takara bio) were grown at 37°C in LB medium (Difco) or on LB agar (Roth) plates supplemented with kanamycin (50 µg/mL), hygromycin (200 µg/mL), or zeocin (50 µg/mL), when required. Smooth (S) and rough (R) variants of *M. abscessus* subsp. *abscessus* CIP104536^T^ (53) and related mutants were grown at 37°C in Middlebrook 7H9 broth (BD Difco) supplemented with 10% oleic acid, albumin, dextrose, catalase (OADC enrichment), 0.2% glycerol, and 0.025% Tyloxapol (Sigma-Aldrich); Mueller Hinton broth (Millipore); in Sauton’s liquid medium (4 g/L asparagine, 0.5 g/L K_2_HPO_4_, 0.5 g/L MgSO_4_, 2 g/L citric acid, 60 g/L glycerol, 0.05 g/L ferric ammonium citrate, 0.0002% ZnSO_4_, adjusted to pH 7.2 with NaOH); on Middlebrook 7H10 agar supplemented with OADC (7H10^OADC^) or on LB agar (Roth) plates containing the appropriate antibiotics. When required kanamycin (250 µg/mL), hygromycin (1 mg/mL), or zeocin (25 µg/mL) or various concentrations of anhydrotetracycline (Sigma-Aldrich) (0, 50, 100, 200, 500, and 1,000 ng/mL) were added to the culture media.

### Construction of Tet-OFF-derived vectors

To control repression of gene expression, we adapted a repressible expression vector based on the Tet-OFF system, reported previously (23, 25). pEN41A-T38S38 and pEN12A-P766 plasmids were kindly provided By D. Schnappinger (Addgene plasmid #9524; http://n2t.net/addgene: 49524; RRID: Addgene_49524). Briefly, the T38 sequences (encoded by *tetR38* with the *Ptb38* promoter sequence), the *tet* operator sequence (*tetO*-4C5G), and genes of interest*—mCherry*, *mmpL3* (*MAB_4508*) fused to an HA-tag—were PCR amplified using the Q5 High-Fidelity DNA Polymerase (New England Biolabs) using pEN12A-P766, pEN41A-T38S38, and pGMCS-mCherry plasmids and *M. abscessus* genomic DNA as templates. Primers were designed to add introduce an HA-tag at the 3′ end of *mmpL3*. Linear fragments were purified on agarose gels (NucleoSpin Gel and PCR Clean-up, Macherey-Nagel). Following the manufacturer’s instructions, In-Fusion SNAP Assembly Master Mix (Takara) reactions were performed to insert these linear fragments into the integrative pMV306 (insertion at the *attL5* mycobacteriophage insertion site in the *glyV* tRNA gene). The resulting plasmids were transformed into Stellar competent cells (Takara Bio) then purified (NucleoSpin Plasmid, Macherey-Nagel) and verified by sequencing. Plasmids were then electroporated into the *M. abscessus* S and/or R variants. To monitor the bacteria *in vivo*, green versions of the resulting plasmids were generated by adding the *mWasabi* coding sequence under the control of the constitutive P_left*_ promoter using the PciI and XbaI restriction sites. The P_left*_ element is a derivative from the P_left_ promoter from mycobacteriophage L5 (54) associated with a ribosomal binding site that increases expression levels (27).

### Generation of an *mmpL3* conditional knockdown mutants

The additional copy of *mmpL3* (under the control of the Tet-OFF system, described above) was first checked in the wild-type S strain by Western blotting, taking advantage of the HA-tag. Unmarked deletion of the endogenous *mmpL3* gene was performed in the merodiploid strain using strategy developed previously (55). Briefly, the pUX1-*katG-mmpL3* was designed to generate an unmarked deletion of 2,982 bp (99.2%) of the open-reading frame. After the two steps of selection of the homologous recombination events, the DNA junctions were PCR amplified and sequenced to confirm the proper genotype of the mutants using primers listed in Table S3 and to validate the generation of S Δ*mmpL3*::c. While this strategy failed in the rough morphotype, the R Δ*mmpL3*::c conditional mutant was obtained by deleting the *mmpL4b* gene (*MAB_4115* c) in S Δ*mmpL3*::c using the pUX1-*katG-mmpL4b*. The unmarked deletion of 2,925 bp (98.7%) of the open-reading frame of *mmpL4b* was confirmed by PCR amplification and sequencing. Thus, the R Δ*mmpL3*::c genotype corresponds to S Δ*mmpL3*::c, Δ*mmpL4b*.

### Growth curves

Potential cytotoxicity of ATc on *M. abscessus* was determined as follows. Pre-cultures in exponential phase were diluted to reach an optical density at 595 nm (OD_595nm_) of 0.05 in 15 mL of 7H9 broth supplemented with 10% OADC, 0.2% glycerol, and 0.025% Tyloxapol, and containing a range of ATc concentrations. Cultures were incubated at 37°C under agitation (80 rpm). OD_595nm_ measurements were performed on a daily basis for 3 days.

Pre-cultures of the Δ*mmpL3*::c mutants in exponential phase were diluted in Mueller-Hinton broth medium to reach an initial OD_595nm_ of 0.01 and dispensed in 96-well microtiter plates in the presence of increasing ATc concentrations (200 µL/well). Plates were incubated at 37°C without agitation, and measurements were taken on a daily basis using a spectrophotometer multimode microplate reader (Tecan Spark 10M; Tecan Group Ltd., Switzerland).

### Colony-biofilm model

*M. abscessus* biofilms were produced based on a recently developed method (42). *M. abscessus* cultures were grown in Middlebrook 7H9 broth supplemented with OADC, 0.2% glycerol, and tyloxapol (0.025%) at 37°C and 100 rpm for 72 h. Cultures were centrifuged at 3,500 rpm for 5 min, washed two times with sterile phosphate buffer saline (PBS), and then diluted to an OD of 0.5 (~1.5 × 10^8^ CFU/mL). Autoclaved black, polycarbonate membranes (diameter, 25 mm; pore size, 0.2 µm; Whatman, Merck, Darmstadt, Germany) were placed on 7H10^OADC^ and inoculated with 20 µL of the bacterial suspension. The membrane-supported biofilms were statically incubated for 48 h at 37°C, transferred onto fresh 7H10^OADC^ containing ATc (50, 100, 200, 500, and 1,000 ng/mL) and incubated at 37°C for an additional 7 days. Every 24 h, the membrane-supported biofilms were imaged and processed using a binocular (ZEISS Axio Zoom.V16, Zeiss, Germany) equipped with a lighting device (Zeiss HXP 200C Zeiss, Germany) at 7× zoom, detecting mWasabi and mCherry fluorescence. On biofilm fluorescence pictures, a straight line was drawn (covering all the diameter of the biofilm), and intensity fluorescence of each pixel was retrieved to draw fluorescence spectra using the ZEN 2 software (Blue Edition).

*MmpL3* conditional mutant biofilms were quantified by CFU counting after taking pictures. Each membrane-supported biofilm was processed for quantifying the number of CFU per membrane at day 7. Biofilms were removed from the plates with sterile clamps, deposited in a 50-mL tube containing 10 mL PBS supplemented with 0.025% tyloxapol and a mixture of 5-mm- and 1-mm-diameter glass beads, vortexed for 15 s, and sonicated once for the S variant and twice for the R variant. The resulting bacterial suspension was diluted in a 10-fold bank dilution and plated on LB agar. Plates were incubated at 37°C for 4 days prior to CFU counting.

### Western blotting

Mycobacterial cultures were grown in Sauton’s broth containing tyloxapol (0.025%) and treated or not with ATc. Bacteria were harvested, centrifuged for 10 min at 3,000 *g* at 4°C, and resuspended in cold PBS containing protease inhibitor cocktail (Sigma-Aldrich). Bacteria were lysed by the addition of 1-mm-diameter glass beads followed by four times 60-s cycle pulses at full speed in a bead-beater device (Mixer Mill MM 301, Retsch). The lysates were collected and protein concentrations assessed using the BCA Protein Assay Reagent kit (ThermoFisher Scientific), according to the manufacturer’s instructions. Equal amounts of proteins (10 µg) were separated by 10% SDS-PAGE and transferred to a PVDF (polyvinylidene fluoride) membrane (Immobilon-P, Merck Millipore). Protein detection was done using rat anti-HA (1:5,000 dilution) antibodies or mouse monoclonal antibodies 32/15, recognizing the Ag85 complex (1:10 dilution) (56). Membranes were then incubated with anti-rat or anti-mouse secondary antibodies conjugated to HRP (horseradish peroxidase) (1:5,000). Signals were detected using HRP reaction (SuperSignal West Femto, ThermoFisher Scientific) on a ChemiDoc MP system (Bio-Rad).

### Whole-cell radiolabeling experiments and lipid analysis

To visualize the ATc-induced changes on the lipid profile, cultures were inoculated at OD = 0.01 in the presence of increasing concentrations of ATc or left untreated for 72 h at 37°C under gentle agitation (100 rpm). As a control, cultures were also treated for 1 h with the indole-2-carboxamide compound 12 (32). Subsequently, metabolic labeling of lipids was performed by adding 1 µCi/mL of [^14^C]acetate (56 mCi/mmol) for an additional 2 h at 37°C. Cells were harvested, delipidated, and further processed to analyze the different lipid fractions, as previously reported (32). The apolar lipid fraction containing TDM was separated on a one-dimensional TLC plate using chloroform/methanol/water (40:8:1, vol/vol/vol). Delipidated cells were further processed to extract the mycolic acids, analyzed by TLC using petroleum ether/acetone (95:5, vol/vol), and exposed to reveal ^14^C-labeled mycolic acid methyl esters.

### Zebrafish maintenance

Zebrafish (*Danio rerio*) were kept and handled in compliance with the guidelines of the European Union for handling laboratory animals and approved by the Direction Sanitaire et Vétérinaire de l’Hérault for the ZEFIX-CRBM zebrafish facility (Montpellier) (registration number C-34–172-39). Handling and experiments were approved by “le ministère de l’enseignement supérieur, de la recherche et de l’innovation” under the reference APAFIS#24406-2020022815234677 V3. Experiments were conducted using the zebrafish *golden* mutants (57), raised in the ZEFIX-CRBM zebrafish facility, and kept on a 12/12-h light/dark cycle. Eggs were obtained by natural spawning, quickly bleached, and incubated at 28.5°C in Petri dishes containing E3 medium (5 mM NaCl, 0.17 mM KCl, 0.33 mM CaCl_2_, 0.33 mM MgSO_4_).

### ATc toxicity assay in zebrafish embryos

To assess the toxicity of ATc in zebrafish, 2 dpf embryos were injected intravenously with an increasing dose of ATc ranging from 1 ng to 100 ng (*n* = 20 embryos/dose). Embryos were placed individually in 48-well plates and monitored daily for 11 days. Clinical signs of toxicity were examined and quantified based on the following criteria: edema, yolk opacification, swim bladder inflation, bent body, heartbeat, blood flow, and response to stimuli, each scored from 0 to 2 (0 = absence, 1 = mild, 2 = severe), yielding a disease score ranging from 0 (no toxicity) to 14 (dead embryo) (Table S4). The dose of 10 ng was chosen for further treatments.

### Zebrafish infection

At 24 hpf, embryos were dechorionated manually with fine tweezers and placed in 60-mm Petri dishes containing E3 at 28.5°C. At 30 hpf, embryos were anesthetized with 0.02% buffered MS222 (Tricaine; ethyl-3-aminobenzoate methanesulfonate salt) and placed in 1.5% agarose plates molded with 0.7 mm slots containing E3 supplemented with 0.02% buffered MS222. Borosilicate glass capillaries (OD = 1 mm; ID = 0.78 mm; 10 cm length) were pulled using a micropipette puller (Sutter instruments P-97). Needles were opened with fine forceps, loaded with 5 µL of the desired inoculum, and supplemented with 0.05% phenol red for microinjection visualization. Microinjection was performed as previously described (7, 39). Embryos were injected in the caudal vein with 1.5 nL of either the R Δ*mmpL3*::c preparation (355 ± 18 CFU) or the PBS control. Injected embryos were then rinsed twice and transferred to 60-mm small Petri dish containing embryo media at 28.5°C. Inoculum was checked *a posteriori* by microinjecting a PBS drop and plating it on 7H10^OADC^.

### Zebrafish ATc treatment

To repress the activity of the Tet-OFF promoter upon infection, half of the embryos from both groups (control and infected) were injected at 1 dpi intravenously with 10 ng of ATc (4 nL of a 2.5-mg/mL solution in PBS) and the other half with the PBS control. Embryos were rinsed twice and randomized over survival assays, CFU counts, and imaging in 48-well plates individually. For survival assays, embryos were monitored daily for 11 days (*n* = 24 per group per experiment). In the absence of a heartbeat, embryos were marked as dead. To quantify bacterial burden at 2 dpi-1 dpt, 3 dpi-2 dpt, and 4 dpi-5 dpt, five embryos from each group were individually disrupted in 50 µL of PBS supplemented with 0.025% of tyloxapol using pellet pestles. Lysates were mixed vigorously with 50 µL of PBS 2% Triton-X-100, sonicated for 30 s, and left for 5 min at room temperature. Ten µL of 10-fold serial dilutions were plated on LB agar supplemented with zeocin (50 µg/mL) and rifampicin (15 µg/mL). Plates were kept at 37°C for 3 days until colonies were countable.

### Fluorescence microscopy imaging

Mycobacterial cultures (OD_600_ = 0.5) expressing fluorescent proteins were 10-fold diluted, and 3 µL of each dilution was spotted on LB agar containing or not ATc and were incubated for 3 days at 37°C. Pictures of bacterial spots and biofilms expressing fluorescent proteins were taken using a binocular (ZEISS Axio Zoom.V16) equipped with a lighting device (Zeiss HXP 200C Zeiss) at 7× zoom detecting the green (mWasabi) and red (mCherry) fluorescence using ZEN 2 (Blue Edition) software.

For zebrafish imaging, embryos were anesthetized with 0.02% buffered MS222 and immobilized with 0.5% low melting agarose in a lateral position in the microinjection molds. Images were acquired with an EVOS M7000 Imaging System every 24 h post-ATc injection for 3 days with the Olympus 2× Objective PlanApo 0.08 NA and the EVOS 10× Objective 0.3NA using the EVOS Light Cube, GFP 2.0. After imaging, embryos were rinsed and transferred back to the 48-well plate with fresh E3. Image processing was done using ZEISS ZEN3.7 software.

### Statistical analyses

All analyses were performed using R (R Core Team, 2017) with R commander (58 – 60) or GraphPad Prism version 9.0.0 for Windows (GraphPad Software, San Diego, California, United States). The normality of data was evaluated using a Shapiro-Wilk test. Descriptive data are cited as median and interquartile range in case of non-normal distribution for each of the variables was calculated. Spearman’s rank correlation coefficient (ρ) was calculated to determine potential relationships between the different concentrations of ATc and the evaluated variables from each experiment. For absolute values of ρ, 0–0.19 is regarded as very weak, 0.2–0.39 as weak, 0.40–0.59 as moderate, 0.6–0.79 as strong, and 0.8–1 as very strong correlation.

Zebrafish survival assays are represented in Kaplan-Meier graphs and analyzed with a log rank test. For determination of bacterial counts, CFU were log_10_ transformed, and the significance between multiple selected groups was determined using one-way ANOVA with Šidák’s multiple comparisons test after validating the normality of the data. A significance level *a priori* was set at α  =  0.05.

## Data Availability

All data generated in this study are available upon request.

## SUPPLEMENTAL MATERIAL

Supplemental material spectrum.02836-23-s0001.pdf Tables S1 to S4; Fig. S1 to S4.

ASM does not own the copyrights to Supplemental Material that may be linked to, or accessed through, an article. The authors have granted ASM a non-exclusive, world-wide license to publish the Supplemental Material files. Please contact the corresponding author directly for reuse.
