# Supplementary material for: Silencing essential gene expression in Mycobacterium abscessus during infection
Source: Microbiol Spectr. 2023 Oct 13;11(6):e02836-23. doi: 10.1128/spectrum.02836-23 (PMC10714871; doi:10.1128/spectrum.02836-23)
Supplement: Supplemental material — Tables S1 to S4; Fig. S1 to S4. [file spectrum.02836-23-s0001.pdf]

Table S1. Strains used in this study.

Strains and Plasmids	Description	Source
Escherichia coli, strain Stellar^{TM}	*E. coli* HST08 strain that provides high transformation efficiency	Takara bio Cat. #636763
M. abscessus CIP104536^{T} Smooth strain(S)	Identifier: ATCC19977^{T}	Laboratoire de Référence des Mycobactéries (IP, France)
M. abscessus CIP104536^{T} Rough strain(R)	Identifier: ATCC19977^{T}	Laboratoire de Référence des Mycobactéries (IP, France)
CIP S::TetOFF,mCherry	Kan^{R} derivative of CIP S in which ATc represses mCherry expression	This work
CIP S::Pleft*,mWasabi-TetOFF,mCherry	Kan^{R} derivative of CIP S in which mWasabi is constitutively expressed and ATc represses mCherry expression	This work
CIP R::Pleft*,mWasabi-TetOFF,mCherry	Kan^{R} derivative of CIP R in which mWasabi is constitutively expressed and ATc represses mCherry expression	This work
CIP S::Pleft*,mWasabi-TetOFF,mmpL3,HA	Zeo^{R} derivative of CIP S in which mWasabi is constitutively expressed and a copy of *mmpL3* gene (HA-tagged) is integrated into the L5 attachment site	This work
CIP S Δ*mmpL3*::c	Zeo^{R} derivative of CIP S in which the native *mmpL3* is deleted, containing a copy of *mmpL3* (HA-tagged) integrated into the L5 attachment site. Expresses constitutively mWasabi	This work
CIP R Δ*mmpL3*::c	Derivative of CIP S Δ*mmpL3*::c in which v*mmpL4b* is deleted to generate a rough variant	This work

Table S2. Plasmids used in this study.

Plasmid	Parent vector/ resisrance	Cloning technique	Source of DNA, Primers, Restriction enzymes	Reference
pEN41A-T38S38	pDO41A<br>Amp^R	-	Source of T38S38 sequence	(Schnappinger et al., 2015)
pEN12A-P766	pDO12A<br>Amp^R	-	Source of P766 promoter	(Schnappinger et al., 2015)
pGMCS-P1-mCherry	pDE43-MCS<br>Str^R	Gateway cloning	Source of *mCherry* sequence	(Boudehen et al., 2022)
pMV306-K	Kan^R			(Stover et al., 1991)
pMV306-K-TetOFF,mCherry	pMV306-K<br>Kan^R	In-Fusion cloning	Linearization of pMV306-K by PvuII<br>PCR using pEN41A-T38R38, pEN12A-P766 and pGMCS-P1-mCherry plasmids as templates with, respectively, TetOFF (F)/Inf TetOFF (R), inf P766 (F)/inf P766 (R2) and Inf XFP Fw/Inf XFP Rv primers and circularization by In-Fusion reaction	This work
L5 *attB*::P_{left*} *mWasabi*	Kan^R			(Kolbe et al., 2020); Addgene ID 69409
pMV306-K-P_{left*} *mWasabi*	pMV361-K-L5 *attB*::P_{left*} *mWasabi*<br>Kan^R	In-Fusion cloning	Linearization of pMV306-K by EcoRV<br>PCR using L5 attB::Pleft* mWasabi as template with p306 P* am Fw/mWasabi Rv primers and circularization by In-Fusion reaction	This work
pMV306-K-P_{left*} *mWasabi*-Tet_{OFF} *mCherry*	pMV306-K-Tet_{OFF} *mCherry*<br>Kan^R	Restriction site cloning	Linearization of pMV306-K-TetOFF,mCherry by XbaI-PciI<br>PCR using Plasmid L5 attB::Pleft* mWasabi as template with inf Pleft am Fw/PciI mFP (R) primers and circularization by T4 DNA Ligase	This work
pMV306-K-Tet_{OFF} *mmpL3-HA*	pMV306<br>Kan^R	In-Fusion cloning	Linearization of pMV306-K by PvuII<br>PCR using pEN41A-T38R38, pEN12A-P766 plasmids and genomic DNA as templates with, respectively, TetOFF (F)/Inf TetOFF (R), Inf P766 (F)/Inf P766 (R2) and inf mmpL3 (F)/inf mmpL3 (R) primers and circularization by In-fusion reaction	This work
pMV306-H-Tet_{OFF} *mmpL3-HA*	pMV306-K-Tet_{OFF} *mmpL3-HA*<br>Hyg^R	In-Fusion cloning	*Hyg* gene amplified using pTEC27H and inf pMV hyg (F2)/inf pMV hyg (R) primers, cloned by In-Fusion reaction in the backbone amplified from	This work

			pMV306-K-TetOFF,mmpL3,HA using pMV atb (Left)/pMV atb (Right) primers	
pMV306-H-P_{left*} *mWasabi*-Tet_{OFF} *mmpL3-HA*	pMV306-H-Tet_{OFF} *mmpL3-HA* Hyg^R	Restriction site cloning	Linearization of pMV306-H-TetOFF,mmpL3 by XbaI-PciI PCR using pMV361-K-L5-attB-Pleft*-mWasabi with inf Pleft am Fw/PciI mFP (R) primers and circularization by T4 DNA Ligase	This work
pMV306-Z-P_{left*} *mWasabi*-Tet_{OFF} *mmpL3-HA*	pMV306-H-Tet_{OFF} *mmpL3-HA* Zeo^R	Restriction site cloning	Digestion of pMV306-Z and pMV306-H-TetOFF,mmpL3,HA by HpaI. Digested fragment encoded Pleft*,mWasabi-TetOFF,mmpL3,HA sequences was cloned into linear pMV306-Z by circularization using T4 DNA ligase	This work
pUX1-*katG-MAB_4106c*	Kan^R			(Daher et al., 2022)
pUX1-*katG-MAB_4508* (*mmpL3*)	pUX1-*katG-MAB_4106c* Kan^R	Restriction site cloning	Linearization of pUX1-KatG-MAB_4106c by PacI-NheI; PCR using genomic DNA as template with mmpL3 KO U PacI (F)/ mmpL3 KO U ovlp (R) and mmpL3 KO D ovlp (F)/mmpL3 KO D NheI (R) primers. Overlapping PCR with the two amplified fragments was done using mmpL3 KO U PacI (F)/ mmpL3 KO D NheI (R) primers was digested with PacI/NheI, cloned into linear pUX1-KatG by circularization using T4 DNA ligase.	This work
pUX1-*katG-MAB_4115c* (*mmpL4b*)	pUX1-*katG-MAB_4106c* Kan^R	Restriction site cloning	Linearization of pUX1-KatG-MAB_4106c by PacI-NheI; PCR using genomic DNA as template with mmpL4b KO U PacI (F)/ mmpL4b KO U ovlp (R) and mmpL4b KO D ovlp (F)/mmpL4b KO D NheI (R) primers. Overlapping PCR with the two amplified fragments was done using mmpL4b KO U PacI (F)/ mmpL4b KO D NheI (R) primers was digested using PacI/NheI, cloned into the linear pUX1-KatG by circularization using T4 DNA ligase.	This work

REFERENCES

Boudehen, Y.-M., Faucher, M., Maréchal, X., Miras, R., Rech, J., Rombouts, Y., et al. (2022). Mycobacterial resistance to zinc poisoning requires assembly of P-ATPase-containing membrane metal efflux platforms. *Nat Commun* 13, 4731. doi: 10.1038/s41467-022-32085-7.

Daher, W., Leclercq, L.-D., Johansen, M. D., Hamela, C., Karam, J., Trivelli, X., et al. (2022). Glycopeptidolipid glycosylation controls surface properties and pathogenicity in Mycobacterium abscessus. *Cell Chem Biol* 29, 910-924.e7. doi: 10.1016/j.chembiol.2022.03.008.

Kolbe, K., Bell, A. C., Prosser, G. A., Assmann, M., Yang, H.-J., Forbes, H. E., et al. (2020). Development and Optimization of Chromosomally-Integrated Fluorescent Mycobacterium tuberculosis Reporter Constructs. *Front Microbiol* 11, 591866. doi: 10.3389/fmicb.2020.591866.

Schnappinger, D., O'Brien, K. M., and Ehrt, S. (2015). "Construction of Conditional Knockdown Mutants in Mycobacteria," in *Mycobacteria Protocols* Methods in Molecular Biology., eds. T. Parish and D. M. Roberts (New York, NY: Springer New York), 151–175. doi: 10.1007/978-1-4939-2450-9_9.

Stover, C. K., de la Cruz, V. F., Fuerst, T. R., Burlein, J. E., Benson, L. A., Bennett, L. T., et al. (1991). New use of BCG for recombinant vaccines. *Nature* 351, 456–460. doi: 10.1038/351456a0.

Table S3. Primers used in this study.

Name	Sequence 5' → 3'
TetOFF (F)	AATATTGGATCACGCCGCGAGC
Inf TetOFF (R)	CTAGATATCCATGGATCCAGCAGCTGGCTAGCGAGTCATGAG
Inf P766 (F)	TCGCGGCGTGATCCAATATTGCTACCAGGCCTAGATCTGG
Inf P766 (R2)	CATGCGGTTGTGAGCGCTCAC
Inf XFP (F)	CGCTCACAACCGCATGGTGAGCAAGGGCGAGGAG
Inf XFP (R)	ATAAGCTTCGAATTCTGCAGTTACTTGTACAGCTCGTCCATGCCG
Inf mmpL3 (F)	CGCTCACAACCGCATGTTCGCCTGGTGGGGCCG
Inf mmpL3 (R)	ATAAGCTTCGAATTCTGCAGCTAAGCGTAATCTGGAACATCGTATGGGTAGA TGCGGCGTCCCTCGCG
Inf pMV hyg (F2)	GTAATACAAGGGGTGTTATAGAGGTCCGCTGTGACACAAG
Inf pMV hyg (R)	CAATTAACCAATTCTGATTACTAGAGGGGGCGTCAGGCG
pMV atb (Left)	AACACCCCTTGTATTACTGTTTATGTAAGCAG
pMV atb (Right)	TCAGAATTGGTTAATTGGTTGTAACACTGGCAG
Inf Pleft am Fw	CTGCTGGATCCATGGATGGCCGCGGTACCAGATCTT
PciI mFP (R)	GGGACATGTCCTGATGCCTGGCAGTCGATCGT
mmpL3 KO U PacI (F)	GAGATTAATTAACGCCCTGGATGTGCTCGAG
mmpL3 KO U ovlp (R)	ATCCCGCGTCGTTAGATGCGCCACCAGGCGAACACAGAATCTC
mmpL3 KO D ovlp (F)	ATTCTGTGTTCGCCTGGTGGCGCATCTAACGACGCGGGATG
mmpL3 KO D NheI (R)	GAGAGCTAGCCTCGTCGGTGCTCTTCCGGT
mmpL4b KO-U-PacI (F)	GAGATTAATTAACCCTTGACGGCGTCGATCAGG
mmpL4b KO U ovlp (R)	AAGACCACTACTCAGAAAGCCGCTCGGCACTCATACCCGGTT
mmpL4b KO D ovlp (F)	GAAACCGGGTATGAGTGCCGAGCGGCTTTCTGAGTAGTGGTCTTGCC
mmpL4b KO D NheI (R)	GAGAGCTAGCGCCAATCCGTCGGCATACGG
seq mmpL3 am (F2)	TGGGAGCGATTGTGAGCCTGG
seq mmpL3 av (R2)	CGCTATCTTGCTGCGCTATCGGA
mmpL4a int (F)	GCGGCCTTGGTCATCGTCG
4114 int (Right)	ACACGCGGAGAACCCAATGG

Table S4: Toxicity of ATc at 11 days post-treatment

Golden+AB
2dpf

Score 0 for absence of symptom; 1 for moderate symptom; 2 for severe symptom

Condition	Embryo	Impaired response to stimuli	Bent body	Abnormal blood flow	Abnormal heart beat	Default in swimming bladder inflation	Yolk opacification	Edema	SCORE
PBS	1	0	0	0	0	0	0	0	0
	2	0	0	0	0	0	0	0	0
	3	0	0	0	0	0	0	0	0
	4	0	0	0	0	0	0	0	0
	5	0	0	0	0	0	0	0	0
	6	0	0	0	0	0	0	0	0
	7	0	0	0	0	0	0	0	0
	8	0	0	0	0	0	0	0	0
	9	0	0	0	0	0	0	0	0
	10	0	0	0	0	0	0	0	0
	11	0	0	0	0	0	0	0	0
	12	0	0	0	0	0	0	0	0
	13	0	0	0	0	0	0	0	0
	14	0	0	0	0	0	0	0	0
	15	0	0	0	0	0	0	0	0
	16	0	0	0	0	0	0	0	0
	17	0	0	0	0	0	0	0	0
	18	0	0	0	0	0	0	0	0
	19	0	0	0	0	0	0	0	0
	20	0	0	0	0	0	0	0	0
ATc 1ng	1	0	0	0	0	0	0	0	0
	2	0	0	0	0	0	0	0	0
	3	0	0	0	0	0	0	0	0
	4	0	0	0	0	0	0	0	0
	5	0	0	0	0	0	0	0	0
	6	0	0	0	0	0	0	0	0
	7	0	0	0	0	0	0	0	0
	8	0	0	0	0	0	0	0	0
	9	0	0	0	0	0	0	0	0
	10	0	0	0	0	0	0	0	0
	11	0	0	0	0	0	0	0	0
	12	0	0	0	0	0	0	0	0
	13	0	0	0	0	0	0	0	0
	14	0	0	0	0	0	0	0	0
	15	0	0	0	0	0	0	0	0
	16	0	0	0	0	0	0	0	0
	17	0	0	0	0	0	0	0	0
	18	0	0	0	0	0	0	0	0
	19	0	0	0	0	0	0	0	0
	20	0	0	0	0	0	0	0	0
ATc 2.5ng	1	0	0	0	0	0	0	0	0
	2	0	0	0	0	0	0	0	0
	3	0	0	0	0	0	0	0	0
	4	0	0	0	0	0	0	0	0
	5	0	0	0	0	0	0	0	0
	6	0	0	0	0	0	0	0	0
	7	0	0	0	0	0	0	0	0
	8	0	0	0	0	0	0	0	0
	9	0	0	0	0	0	0	0	0
	10	0	0	0	0	0	0	0	0
	11	0	0	0	0	0	0	0	0
	12	0	0	0	0	0	0	0	0
	13	0	1	0	0	0	0	0	1
	14	0	0	0	0	0	0	0	0
	15	0	0	0	0	0	0	0	0
	16	0	0	0	0	0	0	0	0
	17	0	0	0	0	0	0	0	0
	18	0	1	0	0	0	0	0	1
	19	0	0	0	0	0	0	0	0
	20	0	0	0	0	0	0	0	0
ATc 5ng	1	0	0	0	0	0	0	0	0
	2	0	0	0	0	0	0	0	0
	3	0	0	0	0	0	0	0	0
	4	0	0	0	0	0	0	1	1
	5	0	0	0	0	0	0	0	0
	6	0	0	0	0	0	0	0	0
	7	0	0	0	0	0	0	0	0
	8	0	1	0	0	0	0	0	1
	9	0	0	0	0	0	0	0	0
	10	0	0	0	0	0	0	0	0
	11	0	0	0	0	0	0	0	0
	12	0	0	0	0	0	1	0	1
	13	0	0	0	0	0	0	0	0
	14	0	0	0	0	0	0	0	0
	15	0	0	0	0	0	0	0	0
	16	0	0	0	0	0	0	1	1
	17	0	0	0	0	0	0	0	0
	18	0	0	0	0	0	0	0	0
	19	0	0	0	0	0	0	0	0
	20	0	0	0	0	0	0	0	0
ATc 10ng	1	0	0	0	0	0	0	0	0
	2	0	0	0	0	0	0	0	0
	3	0	0	0	0	0	0	0	0
	4	0	0	0	0	0	0	1	1
	5	0	0	0	0	0	0	0	0
	6	0	0	0	0	0	0	0	0
	7	0	0	0	0	0	0	0	0
	8	0	0	0	0	1	0	1	2
	9	0	0	0	0	0	0	0	0
	10	0	0	0	0	0	0	0	0
	11	0	0	0	0	0	0	1	1
	12	0	0	0	0	0	0	0	0
	13	0	1	0	0	0	0	0	1
	14	0	0	0	0	1	0	1	2
	15	0	0	0	0	0	0	0	0
	16	0	0	0	0	0	0	0	0
	17	0	0	0	0	0	0	0	0
	18	0	0	0	0	1	0	1	2
	19	0	1	0	0	0	0	0	1
	20	0	0	0	0	0	0	0	0
ATc 20ng	1	2	2	2	2	2	2	2	14
	2	0	2	2	0	2	2	2	10
	3	0	2	2	0	2	2	2	10
	4	1	2	0	0	0	0	2	5
	5	0	1	0	1	1	1	1	5
	6	1	1	1	1	1	1	1	7
	7	0	2	0	0	1	2	0	5
	8	0	0	0	0	2	0	2	4
	9	1	0	0	1	2	1	0	5
	10	0	0	0	0	2	2	2	6
	11	0	0	0	0	1	1	1	3
	12	0	2	0	0	0	0	0	2
	13	0	0	0	0	0	0	0	0
	14	0	0	0	0	0	0	0	0
	15	0	1	0	1	1	1	1	5
	16	0	0	0	0	2	1	2	5
	17	0	1	0	0	1	1	1	4
	18	0	0	0	0	1	1	1	3
	19	0	1	0	1	1	1	1	5
	20	0	0	0	0	0	1	1	2
ATc 50ng	1	2	2	2	2	2	2	2	14
	2	2	2	2	2	2	2	2	14
	3	2	2	2	2	2	2	2	14
	4	1	2	1	2	2	2	2	12
	5	2	2	1	1	2	2	2	12
	6	1	2	1	1	2	1	2	10
	7	0	0	0	0	2	0	2	4
	8	1	2	0	0	2	1	1	7
	9	0	0	0	0	2	1	2	5
	10	0	1	0	0	2	1	2	6
	11	0	0	0	0	1	0	2	3
	12	0	2	1	1	1	1	2	8
	13	0	0	0	0	2	0	2	4
	14	0	1	0	0	2	0	2	5
	15	0	2	0	0	0	0	0	2
	16	0	1	0	0	1	0	1	3
	17	0	0	0	1	1	1	1	4
	18	1	1	1	1	1	1	1	7
	19	0	1	1	1	1	0	1	5
	20	0	1	1	1	1	1	1	6
ATc 100ng	1	2	2	2	2	2	2	2	14
	2	2	2	2	2	2	2	2	14
	3	2	2	2	2	2	2	2	14
	4	2	2	2	2	2	2	2	14
	5	2	2	2	2	2	2	2	14
	6	2	2	2	2	2	2	2	14
	7	2	2	1	1	2	2	2	12
	8	1	2	1	1	1	1	1	8
	9	0	0	0	0	2	2	2	6
	10	0	2	0	0	2	0	0	4
	11	1	1	1	1	1	1	1	7
	12	1	1	1	1	2	2	2	10
	13	2	2	1	1	2	2	2	12
	14	0	1	1	1	2	2	2	9
	15	0	1	1	1	1	0	1	5
	16	0	2	0	0	2	0	0	4
	17	0	1	1	1	1	1	1	6
	18	0	1	1	1	1	1	2	7
	19	1	2	1	1	1	1	1	8
	20	2	0	1	1	2	2	1	9
		0= normal twitching<br>1= slow/extra twitching<br>2= no twitching	0= normal<br>1= slight curve<br>2= very curved	0= normal<br>1= slower or faster<br>2= blockage	0= normal<br>1= slower or faster<br>2= absent = dead embryo	0= nice bubble<br>1= not properly inflated<br>2= collapsed SB	0= no opacification<br>1= less than half of yolk<br>2= more than halk of yolk	0= no edema<br>1= small edema<br>2=big edema	
		Touch with tip			Measure heart beat for 20s then x3 to have bpm				

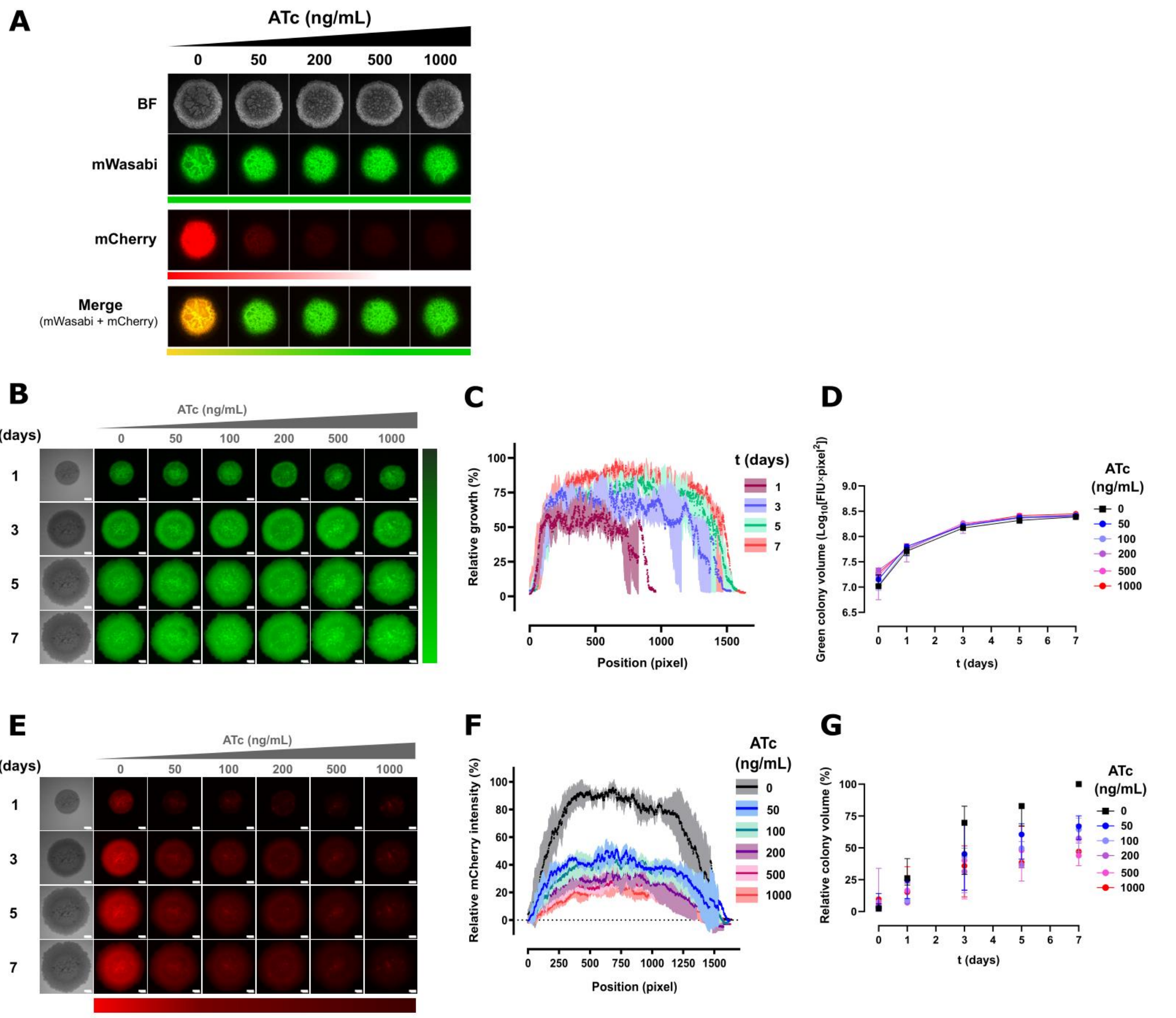

Supplementary Figure 1. Use of dual fluorescent reporter during *M. abscessus* rough biofilm growth. (A) *M. abscessus* CIP R strain, expressing repressible mCherry and constitutive mWasabi proteins, was grown in 7H9 medium at OD=1. Culture was spotted onto LB agar plates supplemented or not with different concentrations of ATc (50, 100, 200, 500, 1000 ng/mL). Plates were incubated 3 days at 37 °C then fluorescent pictures were taken. Biofilm growths of *M. abscessus* CIP R expressing the dual fluorescent reporter were tracked over time. Fluorescent pictures of the biofilms were taken at 1, 3, 5, and 7 days showing **(B)** mWasabi and **(E)** mCherry protein expressions depending on ATc presence (50, 100, 200, 500, 1000 ng/mL) or not. Scale, 2 mm. **(C)** Relative growth of the untreated biofilm (ATc 0) measured by taking the average intensity

levels of the constitutive mWasabi fluorescent protein along the diameter of biofilms. Growth was expressed as % of the seventh day (where 100% is the maximum intensity at day 7). Light colors are corresponding to error bars for each pixel. **(D)** Green colony volume of R biofilms grown in presence of different concentrations of ATc. **(F)** Intensity levels of mCherry fluorescent protein along the diameter of biofilms were retrieved from biofilms growing on different concentration of ATc at day 7. The mCherry intensity was normalized to the control without drug (ATc 0) and expressed as %. **(G)** Red colony volume percentage of R biofilms grown in presence of different concentrations of ATc. Percentages were estimated by considering the red colony volume of S biofilms grown in presence of 0 ng/mL at day 7 as 100%.

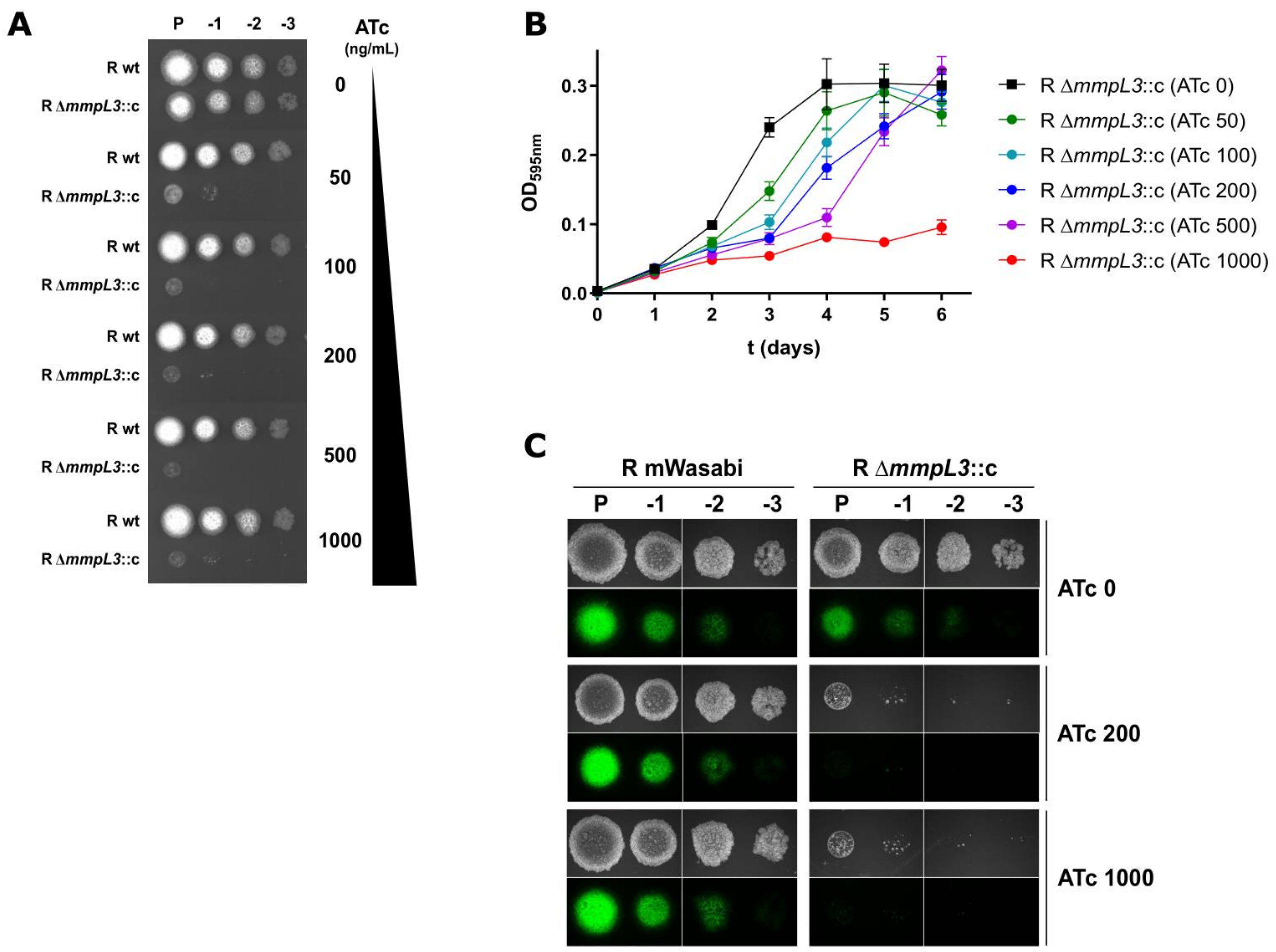

Supplementary Figure 2. *In vitro* characterization of CIP R *M. abscessus mmpL3* conditional knock-down mutant. (A) Parental (R wt) and *mmpL3* conditional mutant (R Δ*mmpL3*::c) cultures were grown to exponential phase and 3 µL of 10-fold serial dilutions were spotted onto LB agar medium supplemented or not with ATc (50, 100, 200, 500, 1000 ng/mL). Plates were incubated at 37 °C. Pictures were taken after 3 days. **(B)** Growth curves of the R Δ*mmpL3*::c exposed to a range of ATc concentrations for 6 days. OD_{600} measurements were taken every day. **(C)** Cultures of green versions of parental (R mWasabi) and R Δ*mmpL3*::c were grown to exponential phase and 3 µL of 10-fold serial dilutions were spotted onto LB agar medium supplemented or not with ATc (200, 1000 ng/mL). Plates were incubated at 37 °C. Fluorescent detection and pictures were taken after 3 days.

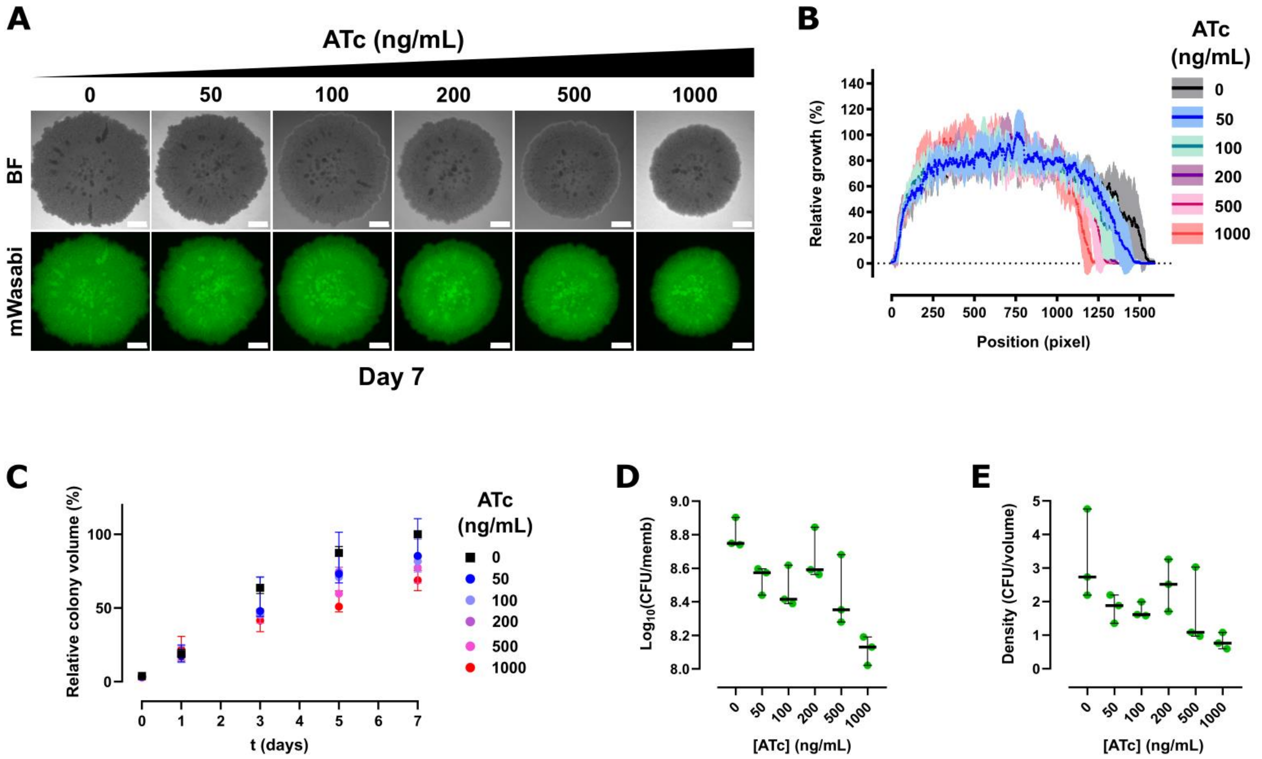

Supplementary Figure 3. Analysis of ATc effect on biofilm of the R *mmpL3* conditional mutant. Analysis was done after 7 days of ATc treatment. **(A)** Development of R Δ*mmpL3*::c biofilm under ATc treatment and visualized by brightfield (BF) and green fluorescent (mWasabi) pictures. **(B)** Intensity levels of mWasabi fluorescent protein along the diameter of biofilms were retrieved from biofilms growing on different concentrations of ATc. The mWasabi intensity was normalized to the control without drug (ATc 0) and expressed as %. Light colors are corresponding to error bars for each pixel. **(C)** Relative colony volume percentage of R Δ*mmpL3*::c biofilms grown in presence of different ATc concentrations at day 0, 1, 3, 5 and 7. Percentages were estimated by considering the green colony volume of biofilms grown in absence of ATc at day 7 as 100%. **(D)** Colony-forming units (CFU) per membrane of R Δ*mmpL3*::c biofilms grown in the presence of different concentrations of ATc at day 7. **(E)** Mycobacterial density (CFU per colony volume unit) of R Δ*mmpL3*::c biofilms grown in the presence of different ATc concentrations at day 7.

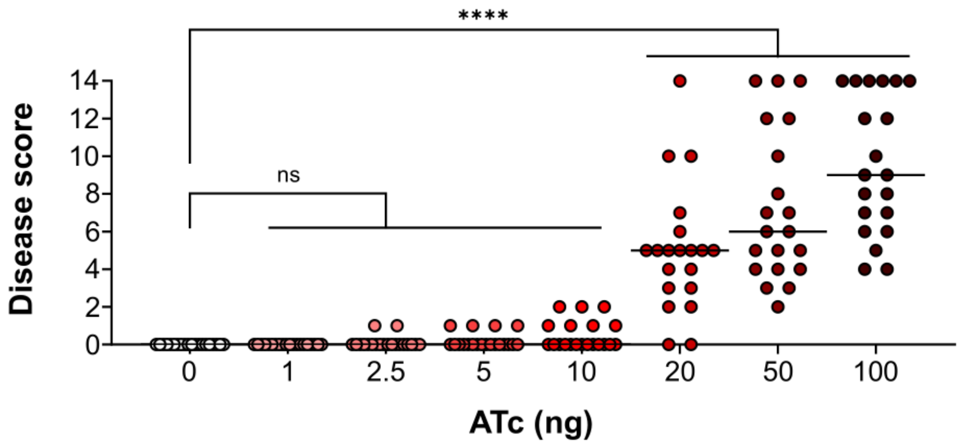

Supplementary Figure 4. Disease score of zebrafish embryos treated intravenously with ATc ranging from 1 ng to 100 ng. Embryos were injected at 2 dpf with ATc ranging from 1 ng to 100 ng. Disease score was determined daily using a series of criteria listed in the Material and Methods. The graph represents the disease score at 11 days post-treatment (dpt). Each dot represents one embryo and the line corresponds to the median. Statistical analysis was done using Kruskal-Wallis test with Dunn's Multiple Comparisons Test.
